# Impact of postmastectomy radiation therapy in T1-2 breast cancer patients with 1-3 positive axillary lymph nodes

**DOI:** 10.18632/oncotarget.17318

**Published:** 2017-04-21

**Authors:** Hang Yin, Yuanyuan Qu, Xiaoyuan Wang, Tengchuang Ma, Haiyang Zhang, Yu Zhang, Yang Li, Siliang Zhang, Hongyu Ma, Enkang Xing, Xueying Liu, Qingyong Xu

**Affiliations:** ^1^ The Department of Breast Radiotherapy, Harbin Medical University Cancer Hospital, Harbin, Heilongjiang Province, China; ^2^ The Department of Internal Medical Oncology, Harbin Medical University Cancer Hospital, Harbin, Heilongjiang Province, China; ^3^ The Department of Nuclear Medicine, Harbin Medical University Cancer Hospital, Harbin, Heilongjiang Province, China; ^4^ The Department of General Surgery, The First Affiliated Hospital of Harbin Medical University, Harbin, Heilongjiang Province, China

**Keywords:** breast neoplasms, surgery, radiotherapy, prognosis, locoregional recurrence

## Abstract

The effect of postmastectomy radiotherapy (PMRT) on T1-2 breast cancer patients with 1-3 positive axillary lymph nodes is controversial up to now. The purpose of this study was to evaluate the impact of postmastectomy radiotherapy for these patients. The prognostic factor effecting locoregional free-survival (LRFS) was also analyzed. In the retrospective clinical data of 1674 eligible patients, survival analysis was performed using the method of Kaplan-Meier and the log-rank test. Cox regression analysis was applied to identify the significant prognostic factors. We found PMRT increased 5-year LRFS (*p*=0.003), but could not improve 5-year disease-free survival or overall survival statistically. For patients without PMRT, multivariate analysis revealed that age, lymph node ratio and molecule subtype were risk factors effecting LRFS. To further analyze the role of PMRT, we grouped all the patients into low risk group (0 or 1 risk factor) and high risk group (2 or 3 risk factors) depending on these risk factors. We found that in low-risk group, PMRT increased only 5-year LRFS (*p*=0.012). However, in high-risk group, PMRT increased both 5-year LRFS (*p*=0.005) and 5-year disease-free survival (*p*=0.033), but could not improve 5-year overall survival statistically. Thus, these data provide the evidence that PMRT could improve LRFS for T1-2 breast cancer patients with 1-3 positive axillary lymph nodes. Additionally, PMRT could improve LRFS and disease-free survival for high risk patients. Age, lymph node ratio and molecule subtype were high risk factors effecting LRFS in our study.

## INTRODUCTION

Breast cancer is one of the most common diagnosed cancers and accounts for 15% of cancer-related death among women in worldwide, with an estimated 1.7 million cases and 521,900 deaths [1]. However, locoregional recurrence (LRR) is still a major obstacle of clinical treatments for prevention of the cancer and outcome after mastectomy [2]. Almost 30% patients present with disease recurrence during follow-up [3].

Several randomized controlled clinical trials found that postmastectomy radiotherapy (PMRT) can improve loco-regional control and overall survival (OS) among patients with advanced T3/T4 primary cancer or with four or more positive lymph nodes [4–6]. So PMRT has been a standard treatment for these patients. PMRT has been also suggested for patients with T1-2 and one to three positive lymph nodes as beneficial but not been widely adopted. A report from the Early Breast Cancer Trialist's Collaborative Group (EBCTCG) showed that patients who received PMRT have a lower 15-year mortality [7]. However, the EBCTCG study was not a random trial. The European Society for Medical Oncology guidelines did not strongly recommend PMRT for all the patients but only recommend for these patients with high risk factors associated with LRR [8]. A recent report from Japan showed that PMRT did not show significant value [9]. Therefore, there is still controversy about the role of PMRT for these patients and the impact of PMRT should be noticed.

This retrospective study reported our institutional data and aimed to evaluate the role of PMRT for T1-2 breast cancer patients with 1-3 positive lymph nodes. The risk factors affecting locoregional free-recurrence survival (LRFS) were also further explored.

## RESULTS

### Survival

**Figure 1:**
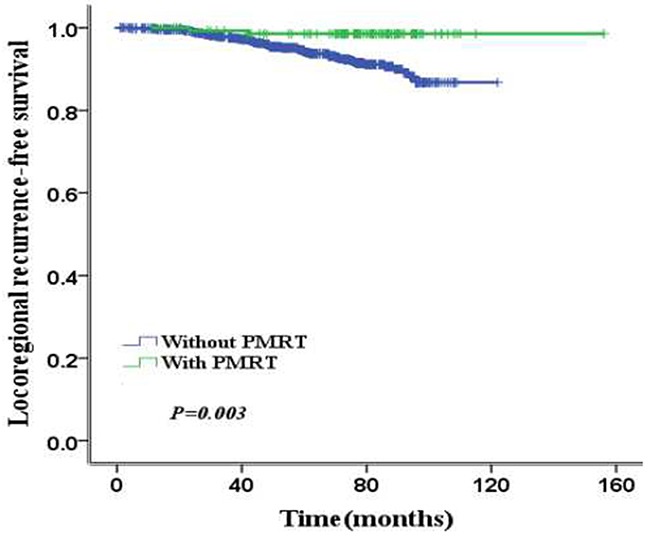
The 5-year LRFS according to PMRT

Median follow-up time was 60 months (range: 9-115). The common data of all the patients were listed in Table 1. There are 87 (5%) patients experienced LRR and the 5-year LRFS was 94.7%. 86 (0.6%) patients without PMRT experienced LRR. 1 (0.6%) patients with PMRT experienced LRR. The 5-year LRFS were 98.5% and 94.2% for patients with PMRT and without PMRT, respectively (*p*=0.003) (Figure 1). The total 5-year disease-free survival (DFS) was 88.9%. The 5-year DFS were 89.4% and 88.7% of patients with PMRT and without PMRT (*p*=0.67). 147 patients died of all patients. There are 137 patients dying from breast cancer and the others are dying of other diseases. The total 5-year OS was 91.1%. For two groups of patients with PMRT and without PMRT, the 5-year OS were 91.6% and 91% (*p*=0.67).

**Table 1:** Patient characteristics and clinical data

Characteristic	Entire value	PMRT(n=149)	Without PMRT(n=1525)	P
Age(years)				
Median	49	46	49	
Range	22-83	25-64	22-83	
Age distribution				<0.001
≤40	180(10.8%)	29(19.5%)	151(9.9%)	
>40	1494(89.2%)	120(80.5%)	1374(90.1%)	
Menopausal status				<0.001
Premenopausal	726 (43.4%)	89(63.3%)	637(41.8%)	
Postmenopausal	948 (56.6%)	60(36.7%)	888(58.2%)	
Tumor classification				<0.001
T1	704 (42.0%)	41(27.6%)	663(43.5%)	
T2	970 (58.0%)	108(72.4%)	862(56.5%)	
Pathology type				0.003
Invasive ductal carcinoma	1480 (88.4%)	119(79.9%)	1361(89.2)	
Lobular carcinoma	134(8.1%)	20(13.4%)	114(7.4%)	
Others	60 (3.5%)	10(6.7%)	50(3.4%)	
Positive lymph nodes				<0.001
1	868 (51.8%)	50(33.6%)	818(53.6%)	
2	449(26.8%)	33(22.1%)	416(27.2%)	
3	357 (21.4%)	66(44.3%)	291(19.2%)	
Ratio of positive nodes				<0.001
≤0.2	1529(91.3%)	113(75.8%)	1416(92.8%)	
>0.2	145(8.7%)	36(24.2%)	109(7.2%)	
Molecular subtypes				<0.001
Luminal A	1108 (66.2%)	45(30.2%)	1063(69.7%)	
Luminal B	127 (7.6%)	30(20.1%)	97(6.4%)	
Her2-enriched	157 (9.4%)	36(24.3%)	121(7.9%)	
Triple-negative	260 (15.5%)	32(21.2%)	228(15.0%)	
Unknown	22 (1.3%)	6(4.2%)	16(1.0%)	
Trastuzumad				0.035
Yes	6	2	4	
No	1668	147	1521	
Hormonal therapy				<0.001
Yes	1222	88(59.1%)	1134(74.4%)	
No	452	61(40.9%)	391(25.6%)	

### Prognostic factors for LRFS

**Figure 2:**
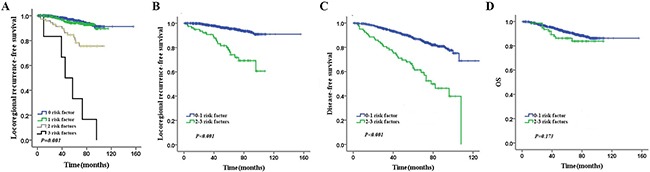
The 5-year LRFS, DFS and OS of patients by risk factor **(A)** The 5-year LRFS of patients with 0, 1, 2, and 3 risk factors. **(B)** The 5-year LRFS of patients with 0-1 and 2-3 risk factors **(C)** The 5-year DFS of patients with 0-1 and 2-3 risk factors **(D)** The 5-year OS of patients with 0-1 and 2-3 risk factors.

On univariate and multivariate analysis, PMRT was independent risk factors for LRFS (*p*=0.003). For patients without PMRT, age (*p*=0.001), lymph node ratio (LNR) (*p*=0.001), positive axillary lymph node (ALN) number (*p*=0.04) and molecule subtype (*p*=0.001) were prognostic risk factors for LRFS on univariate analysis. On multivariate analysis of the patients without PMRT, age (*p*=0.001), LNR (*p*=0.015), and molecule subtype (*p*=0.001) were prognostic risk factors for LRFS (Table 2, 3). Furthermore, we took age, LNR and molecule subtype for grouping analysis, results revealed that the 5-year LRFS rates for the presence of 0, 1, 2, or 3 risk factors were 96%, 93.6%, 84.9% and 33.3%, respectively (*p*=0.001) (Figure 2A). To further analyze the role of PMRT, we grouped all the patients into low risk group (0 or 1 risk factor) and high risk group (2 or 3 risk factors) depending on these risk factors. The 5-year LRFS, DFS and OS of low-risk patients and high-risk patients were 95.4% vs. 79.8% (*p*=0.001), 87.5% vs. 63.2% (*p*<0.001) and 91.3% vs. 86.2% (*p*=0.173), respectively (Figure 2B–2D).

**Table 2:** Univariate analysis of prognostic factors for locoregional recurrence-free survival

Characteristic	Entire cohort			Without PMRT		
	HR	95% CI	*P*	HR	95% CI	*P*
Age, years (< 40 vs. ≥ 40)	0.553	0.343-0.891	0.015	0.44	0.272-0.71	0.001
LNR (≤0.2 vs. >0.2)	2.387	1.367-4.167	0.002	2.519	1.44-4.405	0.001
Positive lymph nodes N(1-2 vs. 3)	1.39	0.869-2.226	0.170	1.65	1.023-2.666	0.040
Tumor size (T1 vs. T2)	1.167	0.564-2.417	0.677	1.23	0.593-2.549	0.588
Lymphatic invasion(negative vs. positive)	0.813	0.375-1.763	0.600	0.803	0.37-1.744	0.580
Menopausal status (Pre vs. Post)	1.5	0.792-2.838	0.213	1.198	0.632-2.274	0.580
Molecule classification						
(luminal B vs. luminal A)	1.382	0.592-3.225	0.454	1.415	0.606-3.307	0.422
(her2-enriched vs. luminal A)	2.089	1.573-2.773	0.001	2.065	1.543-2,764	0.001
(triple negative vs. luminal A)	1.152	0.946-1.403	0.159	1.154	0.947-1.406	0.156
PMRT (yes vs. no)	0.155	0.038-0.629	0.009			

PMRT, postmastectomy radiotherapy; LNR, lymph node ratio; HR, hazard ratio; CI, confidence interval.

**Table 3:** Multivariate analysis of prognostic factors for locoregional recurrence-free survival

Characteristic	Entire cohort			Without PMRT		
	HR	95% CI	*P*	HR	95% CI	*P*
Age, years (< 40 vs. ≥ 40)	0.467	0.289-0.756	0.002	0.427	0.264-0.691	0.001
LNR (≤0.2 vs. >0.2)	2.131	1.15-3.949	0.016	2.178	1.163-4.076	0.015
Positive lymph nodes N(1-2 vs. 3)	1.443	0.85-2.447	0.174	1.325	0.773-2.272	0.306
Molecule classification						
(luminal B vs. luminal A)	1.89	0.877-4.076	0.104	1.434	0.612-3.359	0.407
(her2-enriched vs. luminal A)	2.03	1.513-2.724	0.001	1.985	1.466-2,687	0.001
(triple negative vs. luminal A)	1.183	0.946-1.403	0.100	1.183	0.968-1.444	0.100
PMRT(yes vs. no)	0.117	0.028-0.484	0.003			

### Effect of PMRT

**Figure 3:**
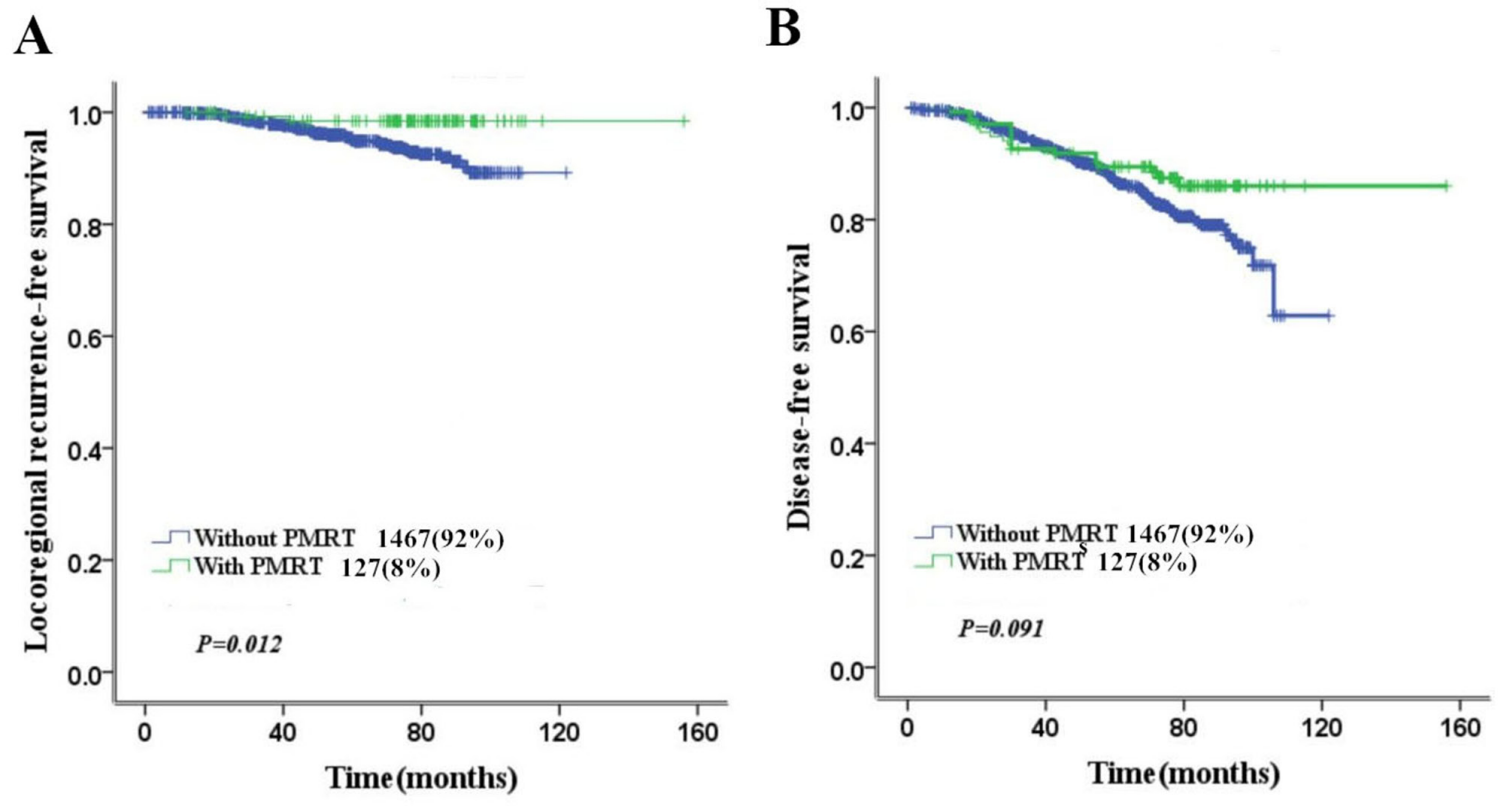
Impact of PMRT on 5-year LRFS and DFS in low-risk patients **(A)** Impact of PMRT on 5-year LRFS in low-risk patients **(B)** Impact of PMRT on 5-year DFS in low-risk patients.

**Figure 4:**
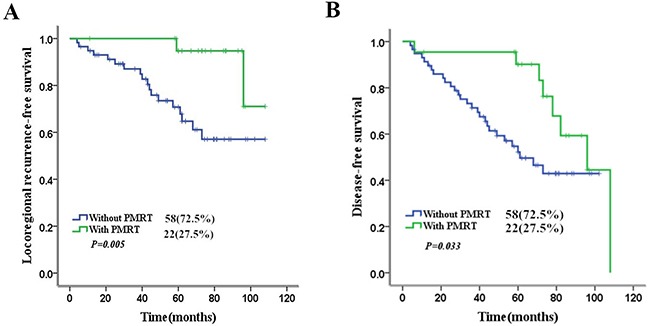
Impact of PMRT on 5-year LRFS and DFS in high-risk patients **(A)** Impact of PMRT on 5-year LRFS in high-risk patients **(B)** Impact of PMRT on 5-year DFS in high-risk patients.

PMRT increased 5-year LRFS from 94.2% to 98.5% (*p*=0.003), but could not improve 5-year DFS (89.4% vs. 88.7%, *p*=0.67) or OS (91.6% vs. 91%, *p*=0.67) statistically (Table 4). In low-risk group, PMRT increased 5-year LRFS from 95% to 98.5% (*p*=0.012), but could not improve 5-year DFS (89.4% vs. 86.9%, *p*=0.091) or 5-year OS (91.8% vs. 91.2%, *p*=0.723) statistically (Figure 3A, 3B). However, in high-risk group, PMRT increased 5-year LRFS from 78% to 96.4% (*p*=0.005) and 5-year DFS from 64.3% to 93% (*p*=0.033), but could not improve 5-year OS (93.1% vs. 86.9%, *p*=0.255) statistically (Figure 4A, 4B).

**Table 4:** Effect of PMRT on survival

Characteristic	With PMRT	Without PMRT	P
All the patients (n)	149	1525	
LRFS (5-year)	98.5%	94.2%	0.003
DFS (5-year)	89.4%	88.7%	0.67
OS (5-year)	91.6	91	0.67
Low risk patients (n)	95	1059	
LRFS (5-year)	98.5	95	0.012
DFS (5-year)	89.4	86.9	0.091
OS (5-year)	91.8	91.2	0.723
High risk patients (n)	54	466	
LRFS (5-year)	96.4	78	0.005
DFS (5-year)	93	64.3	0.033
OS (5-year)	93.1	86.9	0.255

## DISCUSSION

PMRT is a standard adjuvant postoperative therapy for breast cancer patients with T3/T4 primary cancer or with four or more positive lymph nodes. Hamamoto et.al found the locoregional failure risk factors for patients underwent mastectomy without radiotherapy were four or more positive ALNs, pT4, primary tumor larger than 5 cm, lymphatic invasion, and negative hormone receptor status on multivariate analysis. Four or more ALNs was the main risk factor, the others were minor risk factors. The 8-year LRFS were 98%, 97%, 86%, 87%, 88%, 72% and 28% for patients with 0, 1, 2, 3–4 minor risk, only the main risk factor, the main risk with 1-2 minor risk factors and the main risk with 3-4 minor risk factors [10]. Nevertheless, whether T1-2 breast cancer patients with 1 to 3 positive ALNs should receive PMRT or not is still uncertainty. In the report of Nordenskjold, there was little or no influence of PMRT for these patients [11]. However, there are many results of recent studies showed the benefit of PMRT for T1-2 breast patients with 1 to 3 positive ALNs. The Danish Breast Cancer Cooperative Group (DBCG) study, PMRT significantly reduced the 15-year LRR from 27% to 4% (*p*<0.001) and increased the 15-year OS from 48% to 57% with 1-3 ALNs (*p*=0.03) [5]. Additionally, EBCTCG study of a meta-analysis revealed that radiotherapy reduced the 10-year LRR (from 20.3% to 3.8%, log-rank 2*p*<0.0001), 10-year overall recurrence (from 45.7% to 34.2%, RR 0.68, 95% CI 0.57-0.82, log-rank 2*p*=0.00006) and 20-year mortality (from 50.2% to 42.3%, RR 0.80, 95% CI 0.67-0.95, log-rank 2*p*=0.01) [12]. Therefore, PMRT had been strongly consider for T1-2 primary breast tumor patients with 1 to 3 positive ALNs in 2016 American Society of Clinical Oncology [13]. In our study, PMRT also increased 5-year LRFS (98.5% vs. 94.2%, *p*=0.003) which was as same as the previous study.

Whether PMRT can increase the DFS and OS of T1-2 breast cancer patients with 1 to 3 positive ALNs was still unknown. In a recent report of Korea, the study compared the treatment outcomes of patients with T1-2 primary breast cancer and 1-3 positive ALNs between breast conserving surgery (BCS) followed by radiotherapy (BCS+RT) and total mastectomy alone (TM). RT decreased the 5-year cumulative incidence rate of locoregional recurrence from 9.5% to 2.5% (*p* = 0.016) and RT was a relative risk for LRR (*p* = 0.003). RT improved the 5-year DFS (*p* = 0.026) either but didn't increase 5-year OS [14]. Additionally in a report of China, PMRT improved the 10-year LRFS of high risk patients from 63.1% to 98.1% (*p* = 0.002) and 10-year DFS from 57.6 to 81.7% (*p* = 0.007). But the increase of 10-year OS was not observed [15]. Furthermore, a study of Taiwan, 318 T1-2 primary breast cancer patients with 1-3 positive ALNs, reported PMRT decreased LRR from 11% to 3.1% (*p*=0.006) and increased 10-year DFS from 61.3% to 73.8% (*p*=0.001) but didn't increase 5-year OS (*p*=0.239). LNR (*p*=0.003) and LVI (*p*=0.028) were risk factors of local-regional failure by multivariate analysis [16]. Our results also showed PMRT increased 5-year LRFS from 94.2% to 98.5% (*p*=0.003), but did not improve 5-year DFS and OS. However, for high-risk patients, PMRT increased both the 5-year LRFS from 78% to 96.4% (*p*=0.003) and 5-year DFS from 64.3% to 93% (*p*=0.033) which were similar to previous study.

The risk factor of local failure for T1-2 breast cancer patients with 1 to 3 positive ALNs was not clear now by many studies including young age, tumor size, premenopausal status, the number of lymph nodes, molecule subtype, lymphatic vascular invasion (LVI) and LNR. Five National Surgical Adjuvant Breast and Bowel Project Randomized Clinical Trials reported age, tumor size, premenopausal status, the number of lymph nodes were risk factors of LRFS [17]. Additionally, Yang et.al reported ER-negative status and existence of LVI were statistically significance risk factors for locoregional failure [18]. Truong et al. reported LNR>0.20 was closely related to local-regional failure [19]. Additionally, according to a recent study, the risk factors were invasive ductal carcinoma, tumor diameter>2 cm, three lymph nodes, and stage 2b for 1-3 ALNs patients. And these patients can benefit from PMRT [20]. Furthermore, a study from an international breast cancer study group reported that age, PVI and 0-7 uninvolved regional nodes were risk factor of LRR for patients with 1-3 positive ALNs [21]. In our study, age, molecule subtype and LNR were risk factors of LRR as same as previous study. The vessel invasion was not a risk factor which was different from previous study. The reason may be the heterogeneity of the breast cancer and the different period time of follow and the risk factors still need further significant research.

Quantification of the Estrogen receptor (ER), progesterone receptor (PR), human epidermal growth factor receptor2 (HER2) and Ki-67 protein expression was helpful to make clinical decision and predict the outcome [22]. In a recent report of Tianjin China, 1369 T1-2 patients with 1-3 ALNs were included. The molecule subtype was a factor affecting LRR. Among the patients, the Luminal A, Luminal B, Her-2 positive and Triple negative subgroups account for 33.0%, 42.9 %, 11.9 % and 12.2 %, respectively. Molecule subtype was associated with LRR by single and multiple variable analysis (*p*<0.001). The rate of LRR in Her-2 Positive and Triple-negative subtypes were higher (23.3% and 22.2 %) than the Luminal A and Luminal B subtypes (11.5% and 15.0 %) [23]. In our study, molecular subtypes was also a significant predictor for LRFS and Her-2 positive subtype had higher rate of LRR (*p*=0.001). By contrast, a recent report of Europe, ER-positive breast cancer patients had a lower local recurrence than ER-negative patients (9.9% vs. 11.5%, *p* = 0.01) in the first 5 years. After 5 years, ER-positive patients showed higher rate (*p*<0.001) [24]. Furthermore in the report of Shih-Fan Lai, triple-negative breast cancer patients had a higher rate of LRR [25]. Because of the molecule subtype is closely associated with patients' therapeutic results, survival and prognosis, the option of treatment was often according to different molecular subtypes. However, the quantitative influence of subtype on LRR is still in dispute and needs large scale randomized studies [26].

There are some limitations in our study. Firstly, our study was a retrospective single-center study. And the quantity of patients who underwent PMRT was underrepresented in our study. Furthermore, most patients did not receive trastuzumab treatment during that period of time which was different now.

## MATERIALS AND METHODS

### Patients

**Figure 5:**
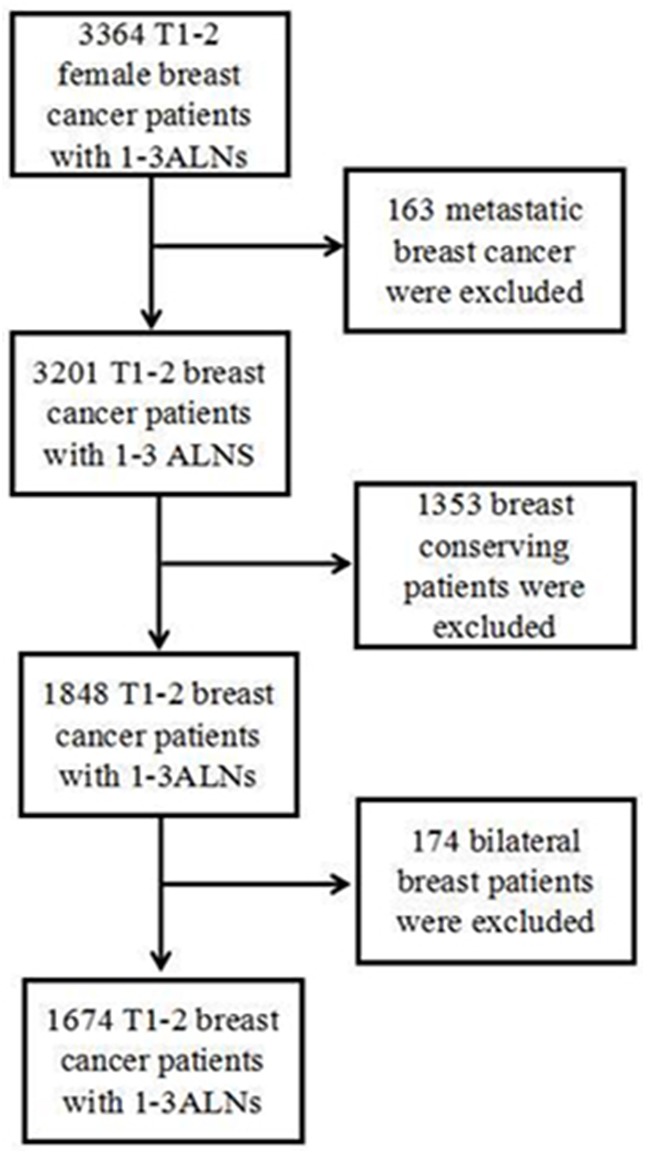
Patients included in the study

We performed a retrospective clinical research include 3364 T1-2 and 1–3 positive ALNs breast cancer patients surgically treated from January2006 to December 2011 at the Harbin Medical University Cancer Hospital. 1674 patients met the criteria and included in our study (Figure 5). The inclusion criteria were unilateral breast cancer female patients, without distant metastasis at first diagnosis and breast conserving patients. This review was approved by our institutional review board. All patients had written permission storage medical information in our hospital database and for research use. In our hospital, the use of PMRT for 1-3 positive lymph nodes was determined by their physicians according to risk factors (e.g. <40 years of age, ratio>0.2, or her-2 rich subtype). The details are shown in Table 1.

All medical records of patients were retrospectively analyzed and evaluated in terms of general characteristics for LRR, DFS and OS. During the follow-up, OS was calculated from the diagnosis date to the last clinical visit or the date of death. LRR was defined as any reappearance of cancer at the ipsilateral chest wall, axilla, internal mammary, supraclavicular or infraclavicular area. Recurrences in other sites were considered as distant metastasis.

### Immunohistochemical Evaluation

ER, PR, HER2, Ki67, and p53 were performed by immunohistochemistry (IHC). HER2-positivity in breast cancer is defined as IHC3+ or a IHC2+ with FISH-positive. Molecular subtypes are divided into Luminal A, Luminal B, HER2-enriched, and Triple-negative subtypes.

### Treatment

All patients received mastectomy and ALN dissection. The median number of ALNs detected was 17 (4 ∼ 51). 1590 (94.9%) patients accepted the chemotherapy, 82 cases(5.1%) for a variety of reasons did not choose to accept systemic chemotherapy. 2 cases is unknown. 149 cases underwent PMRT (8.9%), 1525 (91.1%) did not. The chemotherapy was administered with cyclophosphamide, methotrexate, 5-fluorouracil, cyclophosphamide, doxorubicin or epirubicin. The median cycle of chemotherapy was six (range l ∼ 9). Endocrine therapy was delivered to 1100 Luminal A patients and 122 Luminal B patients with the median medication time at 55 months (range 26-78 months), mainly by antiestrogens and aromatase inhibitors. Premenopausal patients were treated with tamoxifen, and postmenopausal women were treated with tamoxifen or an aromatase inhibitor.

In radiation, 4 or 8 MV photon beams were used. The bolus was not used in these patients. The radiation dose was 50Gy (range: 50- 60Gy) at 1.8 to 2Gy per fraction (25-26 fractions). Radiation targets covered the ipsilateral chest wall and the supraclavicular area of all the patients. The axilla area was covered in 9 patients and internal mammary nodes covered in 14 patients. Six patients of HER2-rich received trastuzum. Two of six patients received PMRT. Four of six patients did not receive PMRT.

### Follow up

All the patients received follow-up every 3 or 6 months by medical review, phone call, or letter. The follow up defined as from the first day after operation. The main aim of our study was the LRR, DFS, and OS. Loco-regional recurrence (LRR) was defined as recurrence of tumor at the ipsilateral chest wall, axilla, internal mammary, supraclavicular or infraclavicular area. The diagnosed of LRR was confirmed by histology.

### Statistical method

Statistical analyses used SPSS 21.0. The prognostic factors affecting LRR, DFS and OS were evaluated by Kaplan-Meier method. Log rank test was used to compare two survival curves. *P*<0.05 was defined as statistical significance. Cox regression analysis was used to determine significant clinically prognostic factors.

## References

[1] Torre LA, Bray F, Siegel RL, Ferlay J, Lortet-Tieulent J, Jemal A (2015). Global cancer statistics, 2012. CA Cancer J Clin.

[2] Clarke M, Collins R, Darby S, Davies C, Elphinstone P, Evans V, Godwin J, Gray R, Hicks C, James S, MacKinnon E, McGale P, McHugh T (2005). Effects of radiotherapy and of differences in the extent of surgery for early breast cancer on local recurrence and 15-year survival: an overview of the randomised trials. Lancet.

[3] Saphner T, Tormey DC, Gray R (1996). Annual hazard rates of recurrence for breast cancer after primary therapy. J Clin Oncol.

[4] Poortmans P (2007). Evidence based radiation oncology: breast cancer. Radiother Oncol.

[5] Overgaard M, Nielsen HM, Overgaard J (2007). Is the benefit of postmastectomy irradiation limited to patients with four or more positive nodes, as recommended in international consensus reports? A subgroup analysis of the DBCG 82 b&c randomized trials. Radiother Oncol.

[6] Van de Steene J, Soete G, Storme G (2000). Adjuvant radiotherapy for breast cancer significantly improves overall survival: the missing link. Radiother Oncol.

[7] Liljegren G, Holmberg L, Bergh J, Lindgren A, Tabár L, Nordgren H, Adami HO (1999). 10-Year results after sector resection with or without postoperative radiotherapy for stage I breast cancer: a randomized trial. J Clin Oncol.

[8] Aebi S, Davidson T, Gruber G, Cardoso F, ESMO Guidelines Working Group (2011). Primary breast cancer: ESMO Clinical Practice Guidelines for diagnosis, treatment and follow-up. Ann Oncol.

[9] Nagao T, Kinoshita T, Tamura N, Hojo T, Morota M, Kagami Y (2013). Locoregional recurrence risk factors in breast cancer patients with positive axillary lymph nodes and the impact of postmastectomy radiotherapy. Int J Clin Oncol.

[10] Hamamoto Y, Ohsumi S, Aogi K, Shinohara S, Nakajima N, Kataoka M, Takashima S (2014). Are there high-risk subgroups for isolated locoregional failure in patients who had T1/2 breast cancer with one to three positive lymph nodes and received mastectomy without radiotherapy?. Breast Cancer.

[11] Nordenskjold AE, Fohlin H, Albertsson P, Arnesson LG, Chamalidou C, Einbeigi Z, Holmberg E, Nordenskjold B, Karlsson P, Swedish Western and Southeastern Breast Cancer Groups (2015). No clear effect of postoperative radiotherapy on survival of breast cancer patients with one to three positive nodes: a population-based study. Ann Oncol.

[12] McGale P, Taylor C, Correa C, Cutter D, Duane F, Ewertz M, Gray R, Mannu G, Peto R, Whelan T, Wang Y, Wang Z, Darby S, EBCTCG (Early Breast Cancer Trialists' Collaborative Group) (2014). Effect of radiotherapy after mastectomy and axillary surgery on 10-year recurrence and 20-year breast cancer mortality: meta-analysis of individual patient data for 8135 women in 22 randomised trials. Lancet.

[13] Recht A, Comen EA, Fine RE, Fleming GF, Hardenbergh PH, Ho AY, Hudis CA, Hwang ES, Kirshner JJ, Morrow M, Salerno KE, Sledge GW, Solin LJ (2016). Postmastectomy Radiotherapy: An American Society of Clinical Oncology, American Society for Radiation Oncology, and Society of Surgical Oncology Focused Guideline Update. J Clin Oncol.

[14] Kim SW, Chun M, Han S, Jung YS, Choi JH, Kang SY, Jang H, Jo S (2016). Comparison of Treatment Outcomes between Breast Conserving Surgery Followed by Radiotherapy and Mastectomy Alone in Patients with T1-2 Stage and 1-3 Axillary Lymph Nodes in the Era of Modern Adjuvant Systemic Treatments. PLoS One.

[15] Wu SG, He ZY, Li FY, Wang JJ, Guo J, Lin Q, Guan XX (2010). The clinical value of adjuvant radiotherapy in patients with early stage breast cancer with 1 to 3 positive lymph nodes after mastectomy. Chin J Cancer.

[16] Huang CJ, Hou MF, Chuang HY, Lian SL, Huang MY, Chen FM, Fu OY, Lin SF (2012). Comparison of clinical outcome of breast cancer patients with T1-2 tumor and one to three positive nodes with or without postmastectomy radiation therapy. Jpn J Clin Oncol.

[17] Taghian A, Jeong JH, Mamounas E, Anderson S, Bryant J, Deutsch M, Wolmark N (2004). Patterns of locoregional failure in patients with operable breast cancer treated by mastectomy and adjuvant chemotherapy with or without tamoxifen and without radiotherapy: results from five National Surgical Adjuvant Breast and Bowel Project randomized clinical trials. J Clin Oncol.

[18] Yang PS, Chen CM, Liu MC, Jian JM, Horng CF, Liu MJ, Yu BL, Lee MY, Chi CW (2010). Radiotherapy can decrease locoregional recurrence and increase survival in mastectomy patients with T1 to T2 breast cancer and one to three positive nodes with negative estrogen receptor and positive lymphovascular invasion status. Int J Radiat Oncol Biol Phys.

[19] Truong PT, Woodward WA, Thames HD, Ragaz J, Olivotto IA, Buchholz TA (2007). The ratio of positive to excised nodes identifies high-risk subsets and reduces inter-institutional differences in locoregional recurrence risk estimates in breast cancer patients with 1-3 positive nodes: an analysis of prospective data from British Columbia and the M. D. Anderson Cancer Center. Int J Radiat Oncol Biol Phys.

[20] Cihan YB, Sarigoz T (2016). Role of postmastectomy radiation therapy in breast cancer patients with T1-2 and 1-3 positive lymph nodes. Onco Targets Ther.

[21] Karlsson P, Cole BF, Chua BH, Price KN, Lindtner J, Collins JP, Kovacs A, Thurlimann B, Crivellari D, Castiglione-Gertsch M, Forbes JF, Gelber RD, Goldhirsch A, Gruber G (2012). Patterns and risk factors for locoregional failures after mastectomy for breast cancer: an International Breast Cancer Study Group report. Ann Oncol.

[22] Voduc KD, Cheang MC, Tyldesley S, Gelmon K, Nielsen TO, Kennecke H (2010). Breast cancer subtypes and the risk of local and regional relapse. J Clin Oncol.

[23] Shen H, Zhao L, Wang L, Liu X, Liu X, Liu J, Niu F, Lv S, Niu Y (2016). Postmastectomy radiotherapy benefit in Chinese breast cancer patients with T1-T2 tumor and 1-3 positive axillary lymph nodes by molecular subtypes: an analysis of 1369 cases. Tumour biol.

[24] Colleoni M, Sun Z, Price KN, Karlsson P, Forbes JF, Thürlimann B, Gianni L, Castiglione M, Gelber RD, Coates AS, Goldhirsch A (2016). Annual Hazard Rates of Recurrence for Breast Cancer During 24 Years of Follow-Up: Results From the International Breast Cancer Study Group Trials I to V. J Clin Oncol.

[25] Lai SF, Chen YH, Kuo WH, Lien HC, Wang MY, Lu YS, Lo C, Kuo SH, Cheng AL, Huang CS (2016). Locoregional Recurrence Risk for Postmastectomy Breast Cancer Patients With T1-2 and One to Three Positive Lymph Nodes Receiving Modern Systemic Treatment Without Radiotherapy. Ann Surg Oncol.

[26] Tramm T, Kyndi M, Myhre S, Nord S, Alsner J, Sørensen FB, Sørlie T, Overgaard J (2014). Relationship between the prognostic and predictive value of the intrinsic subtypes and a validated gene profile predictive of loco-regional control and benefit from post-mastectomy radiotherapy in patients with high-risk breast cancer. Acta Oncol.

